# To the Editors of the Medico-Chirurgical Journal & Review

**Published:** 1817-05

**Authors:** G. N. Hill

**Affiliations:** Chester


*To the Editors of the Medico-Chirurgical Journal & Review.*

GENTLEMEN,

YOUR last Number contains, at p. 217, some remarks on Mr. Earle's and Mr. Brodie's cases of "Contractions succeeding ulcerations of the skin;" that operated upon by the latter gentleman, and forming the third recorded in the paper, was contraction from a burn of the neck, which Mr. E. says, is doubly interesting on account of its being the first in which the "cicatrix was removed for a contraction of the neck." On this you justly remark, "It may be so; but the removal of a contraction itself by dividing the cicatrix, is fresh in our memories, though we cannot call to mind the periodical publication in which the case is related, and a plate given of the parts anterior and subsequent to the operation." The periodical work in which I published the case with a plate, was the Edin. Med. and Surg. Journal for April 1811.

I will take the liberty of trespassing on your time and pages farther to observe, that I presume no surgeon will contend that any of the contractions from burns, not even the axillary, or popliteal, surpass those of the neck and face in serious extent of mischief, and lasting evil, unless removed by surgical assistance. With respect to your remark, that Mr. E. "has established a very useful fact in surgery," far be it from me to aim at throwing a shade of doubt on the justness of your opinion; but from the history of the case related as above, and its fortunate termination, with others that have occurred to me since, I cannot resist the temptation of offering a remark or two before closing this letter. I merely ask for information. What must have been the result of dissecting off all the scorched skin and muscles of the face and front of the neck? Would such a procedure have been certainly followed by the growth of new muscular fibres, cellular substance, and healthy free integuments? Where skin is burnt, and covering bones with but little subjacent muscular substance, what is to supply the place of the dissected cicatrix? Experience has confirmed the truth, that complete division of the hardest parts is succeeded by abatement of rigidity, and ultimate approximation to nearly healthy texture and appearance, care being taken that the division of the parts contracted, be so perfect as to liberate the whole of the previously locked up surrounding and subjacent muscles and cellular membrane; this being done, and the moving powers set free, their due action after long suspension is restored, Nature kindly renders her assistance, and restoration is commonly more perfect than those unmindful of what Nature will effect can readily believe.

But I well know, that where tendon or ligament is bound down with a horny cicatrix, it is often good practice to remove such part of it as will enable us to give perfect liberty to a finger, &c.

Can we remove a cicatrix of some inches in dimensions, without having the space covered with a new cicatrix, which will infallibly form as great an impediment to the necessary and free action of the neighbouring parts as the old one did?

I am, &c.

G. N. HILL.

Chester, March 17, 1817.

